# Cortical Terminations of the Inferior Fronto-Occipital and Uncinate Fasciculi: Anatomical Stem-Based Virtual Dissection

**DOI:** 10.3389/fnana.2016.00058

**Published:** 2016-05-24

**Authors:** Janice Hau, Silvio Sarubbo, Guy Perchey, Fabrice Crivello, Laure Zago, Emmanuel Mellet, Gaël Jobard, Marc Joliot, Bernard M. Mazoyer, Nathalie Tzourio-Mazoyer, Laurent Petit

**Affiliations:** ^1^Groupe d’Imagerie Neurofonctionnelle, Institut des Maladies Neurodégénératives – UMR 5293, CNRS, CEA University of BordeauxBordeaux, France; ^2^Division of Neurosurgery, Department of Neurosciences, “S. Chiara” HospitalTrento, Italy; ^3^Structural and Functional Connectivity Lab, Division of Neurosurgery, “S. Chiara” HospitalTrento, Italy

**Keywords:** white matter, diffusion tensor imaging, tractography, human brain anatomy, uncinate fasciculus, inferior fronto-occipital fasciculus

## Abstract

We combined the neuroanatomists’ approach of defining a fascicle as all fibers passing through its compact stem with diffusion-weighted tractography to investigate the cortical terminations of two association tracts, the inferior fronto-occipital fasciculus (IFOF) and the uncinate fasciculus (UF), which have recently been implicated in the ventral language circuitry. The aim was to provide a detailed and quantitative description of their terminations in 60 healthy subjects and to do so to apply an anatomical stem-based virtual dissection, mimicking classical post-mortem dissection, to extract with minimal *a priori* the IFOF and UF from tractography datasets. In both tracts, we consistently observed more extensive termination territories than their conventional definitions, within the middle and superior frontal, superior parietal and angular gyri for the IFOF and the middle frontal gyrus and superior, middle and inferior temporal gyri beyond the temporal pole for the UF. We revealed new insights regarding the internal organization of these tracts by investigating for the first time the frequency, distribution and hemispheric asymmetry of their terminations. Interestingly, we observed a dissociation between the lateral right-lateralized and medial left-lateralized fronto-occipital branches of the IFOF. In the UF, we observed a rightward lateralization of the orbito-frontal and temporal branches. We revealed a more detailed map of the terminations of these fiber pathways that will enable greater specificity for correlating with diseased populations and other behavioral measures. The limitations of the diffusion tensor model in this study are also discussed. We conclude that anatomical stem-based virtual dissection with diffusion tractography is a fruitful method for studying the structural anatomy of the human white matter pathways.

## Introduction

Both inferior fronto-occipital (IFOF) and uncinate (UF) fasciculi are crucial for the ventral intra-hemispheric transfer of information between the frontal cortex and the occipital, temporal and parietal cortices, and knowing their cortical terminations is fundamental for understanding their role in mediating language semantics (Turken and Dronkers, 2011; Duffau, 2015). The earliest description of the IFOF dates back to Burdach (1819–1826) who described direct fronto-occipital connections although he misattributed them to the inferior longitudinal fasciculus. The UF was described even earlier by Reil (Reil, 1809), characterized as the hooked-shape fibers behind the insula. While the anatomical description of the UF has remained stable (connecting fronto-orbital cortices with the temporo-polar cortex), there have been some inconsistencies surrounding the IFOF’s anatomical course and terminations (Dejerine and Dejerine-Klumpke, 1895; Trolard, 1906; Curran, 1909; Crosby et al., 1962) due to its complex anatomy.

Only recently have *post-mortem* dissection studies begun to investigate the precise terminations of white matter pathways, developing updated techniques to improve precision in locating and identifying their cortical fiber terminations (Martino et al., 2011). A clearer picture of the IFOF’s precise cortical terminations is starting to emerge. In particular, two recent dissection studies have provided in-depth descriptions of its anterior (Sarubbo et al., 2013) and posterior (Martino et al., 2010) terminations. Although less recent, Ebeling and von Cramon (1992) meticulous dissection study on the anatomy of the uncinate fasciculus provides a detailed description of its anatomical projections within the frontal and temporo-mesial areas that elaborates on earlier descriptions. While these dissection studies confirmed the conventional definition of the respective tracts, they also revealed termination territories in more detail and beyond those previously described, suggesting more expansive definitions are needed. Previous dissection work indicates that there is some inter-individual variability in the tract projections (Martino et al., 2010). This no doubt contributes to the inconsistencies in the cortical terminations observed in the IFOF and to a limited extent the UF. Tractography with diffusion-weighted imaging enables the *in vivo* anatomical study of fiber pathways in large samples. To our knowledge, since the seminal single-subject tractography study of Catani (Catani et al., 2002), only one tractography study has examined the cortical terminations of the IFOF in a group (20 subjects) confirming some of the more extensive projections described inconsistently thus far in the literature, in particular within the parietal lobe (Caverzasi et al., 2014). There has been no detailed study of the UF terminations using tractography (Von Der Heide et al., 2013).

**FIGURE 1:**
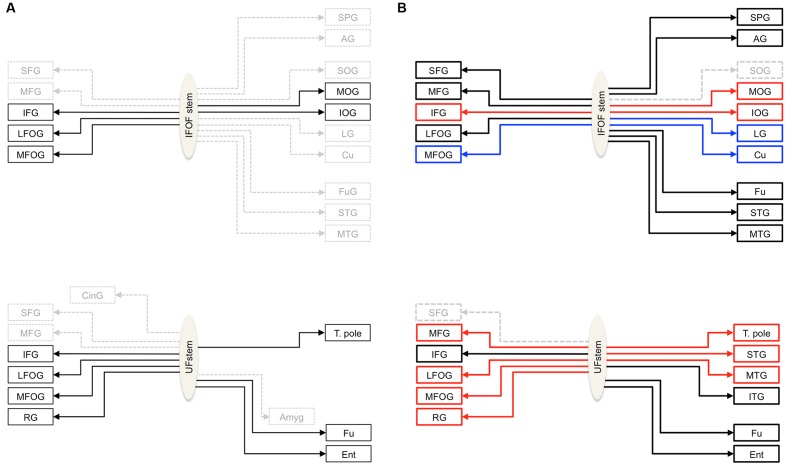
**(A)** Schemas summarizing the cortical termination territories of the IFOF (top) and UF (bottom) in the literature. The cortical territories corresponding to their conventional definitions are shown by solid black arrows and dashed gray arrows show the debated cortical territories. **(B)** Schemas representing the cortical termination territories of the IFOF (top) and UF (bottom) based on our stem-based tractography study. Solid lines represent the confirmed cortical territories and the regions that remain questionable are represented by dashed lines. The significant left lateralized projections are shown in blue and the right lateralized projections in red. Abbreviations: SFG, MFG, IFG, superior, middle and inferior frontal gyri; LFOG, MFOG, lateral and medial fronto-orbital gyri; RG, rectus gyrus; SOG, MOG, IOG, superior, middle and inferior occipital gyri; Cu, cuneus; LG, lingual gyrus; T. pole, temporal pole; STG, MTG, ITG, superior, middle and inferior temporal gyri; Fu, fusiform gyrus; SPG, superior parietal gyrus; AG, angular gyrus; Ent, entorhinal gyrus.

**Figure 1A** summarizes the consensus coming from both *post-mortem* dissection and *in vivo* tractography studies, that the IFOF connects the ventral occipital cortex with the inferior frontal and fronto-orbital cortices (Dejerine and Dejerine-Klumpke, 1895; Trolard, 1906; Curran, 1909; Crosby et al., 1962; Catani and Thiebaut de Schotten, 2008; Martino et al., 2010; Zhang et al., 2010; Sarubbo et al., 2013; Caverzasi et al., 2014; Forkel et al., 2014). But, IFOF projections to the superior and middle frontal (Sarubbo et al., 2013; Caverzasi et al., 2014), medial occipital (i.e., the lingual gyrus and cuneus) (Lawes et al., 2008; Martino et al., 2011; Caverzasi et al., 2014; Forkel et al., 2014), temporo-basal (Crosby et al., 1962; Catani and Thiebaut de Schotten, 2008; Lawes et al., 2008; Martino et al., 2011; Caverzasi et al., 2014) and parietal (Curran, 1909; Martino et al., 2010; Sarubbo et al., 2013; Caverzasi et al., 2014; Forkel et al., 2014) regions are inconsistently observed and their inclusion in its definition is debated. The consensus on the UF is that it connects, along with the fronto-orbital and temporo-polar cortices, ventral temporal areas (Ebeling and von Cramon, 1992; Catani and Thiebaut de Schotten, 2008). Questions also remain concerning the UF terminations, in particular concerning its projections to the amygdala (Klinger and Gloor, 1960; Ebeling and von Cramon, 1992; Croxson et al., 2005; Thiebaut de Schotten et al., 2012) and cingulate gyrus (Ebeling and von Cramon, 1992; Thiebaut de Schotten et al., 2012) posteriorly and to the superior and middle frontal gyri, anteriorly (Dejerine and Dejerine-Klumpke, 1895; Kier et al., 2004; Thiebaut de Schotten et al., 2012). A common understanding of the anatomy of these tracts is needed.

In order to study these open questions of whether a particular cortical region belongs to the IFOF or UF, we adopted the approach of neuroanatomists who first locate the stem, which is the point of passage where all fibers pass through, as an anatomical reference to identify the tract. We therefore performed an anatomical stem-based virtual dissection to extract these two bundles from whole-brain tractography datasets in a large cohort of 60 healthy subjects. Starting from widely accepted anatomical evidence regarding the locations of the IFOF and UF stems, we exposed their respective stems and manually delineated single regions of interest (ROI) around each of them. This approach enables us to minimize *a priori* on their terminations and provide the complete set of cortical terminations of IFOF and UF, as well as their variability and their asymmetries.

## Materials and Methods

### Image Acquisition

Diffusion-weighted images were previously acquired for 60 healthy right-handed (30 female, mean age = 30.1, age range = 20–53) belonging to the BIL&GIN database (Brain Imaging of Lateralization by the Groupe d’Imagerie Fonctionnelle; Mazoyer et al., 2016). All the subjects gave written informed consent to participate in the study, which was approved by the local ethics committee (*CCPRB Basse-Normandie*). Imaging was performed on a Philips Achieva 3 Tesla MRI scanner using a single-shot spin-echo echo-planar sequence with 21 non-collinear diffusion gradient directions (*b* = 1000 s/mm^2^). Seventy axial slices parallel to the AC-PC plane were acquired from the bottom of the cerebellum to the vertex. Imaging parameters were as follows: TR = 8500 ms, TE = 81 ms, angle = 90°, SENSE reduction factor = 2.5, FOV 224 mm, acquisition matrix 112 × 112, 2 mm × 2 mm × 2 mm isotropic voxel. The series of 21 directions was acquired twice by reversing the gradients’ polarity, for a total of 42 diffusion-weighted volumes. To improve the signal-to-noise ratio, a second series of 42 volumes was acquired leading to a total acquisition time of 15 min 30 s.

### Whole-Brain Tractography

**FIGURE 2:**
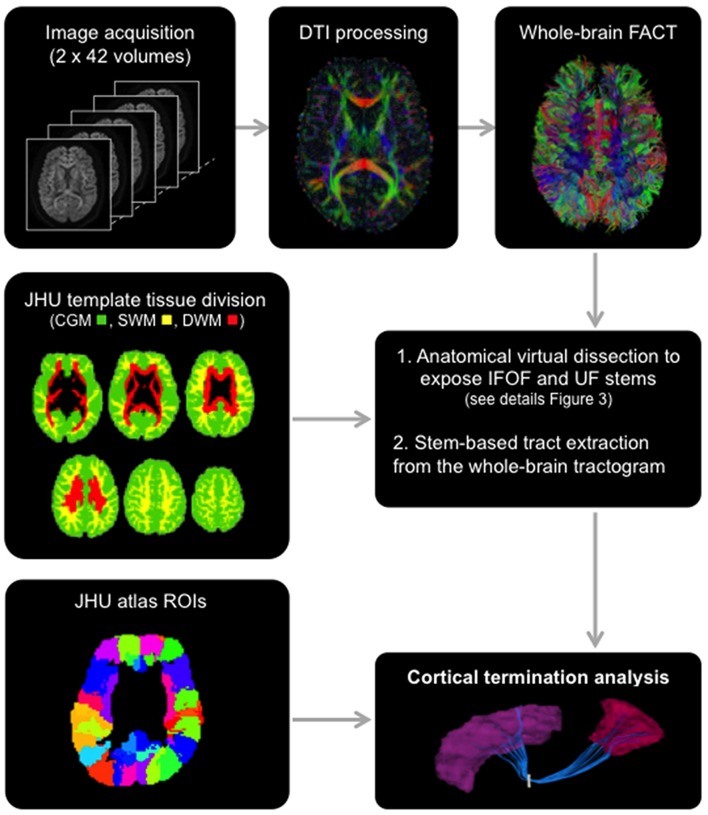
**General scheme of the different steps involved in the study from data acquisition to the extraction of the tracts using the stem-based approach to tract connectivity analysis.** The temporal lobe from the JHU atlas was split to create its gray and superficial white matter pole (shown) and corresponding superior, middle and inferior divisions. The three large divisions, cortical gray matter (green), superficial white matter (yellow) and deep white matter (red), were created by combining regions of the JHU atlas, and used to select the different sub-types of streamlines.

The raw diffusion images were corrected for eddy current distortion using the FMRIB Software Library (Smith et al., 2004) and processed with the Diffusion Toolkit software package to obtain the local tensor orientation estimates and fractional anisotropy maps and perform fiber tracking (**Figure 2**, top row). Deterministic whole-brain fiber tracking was performed in the native space of each subject using the Fiber Assignment by Continuous Tracking algorithm (Mori et al., 1999) with stopping criteria of 0.2 fractional anisotropy and a 45° angle threshold. Tracking was initiated by seeding from all voxels in the volume to generate the streamlines. This produced a 3D reconstruction of streamlines in the whole brain, namely a tractogram, which can then be segmented into anatomically defined bundles.

### Template and Regions of Interest

The Johns Hopkins University (JHU) template (Oishi et al., 2009), containing 176 pre-segmented regions, was modified to create an additional temporal pole region in the standard space of the template. The superior, middle and inferior temporal regions [gyri and superficial white matter (SWM)] were split in two: the temporal pole (anterior to the anterior commissure) and their respective superior, middle and inferior temporal regions (posterior to the anterior commissure) for the left and right hemispheres. In addition, we created three large divisions (**Figure 2**) representing the cortical gray matter (CGM), comprising all cortical areas of the template, deep white matter (DWM), comprising all DWM areas of the template and superficial white matter (SWM), comprising white matter areas associated with the gyral regions (i.e., situated between the cortex and the DWM). The modified JHU template and tissue division ROIs were warped to the native space of each subject using ANTS (Avants et al., 2011) linear and non-linear registration. For the cortical terminations analysis, the modified template was modally dilated once to include the interface between the gray and white matter.

### Stem-Based Anatomical Virtual Dissection of IFOF and UF

Both IFOF and UF were extracted by performing a virtual dissection in each hemisphere of the 60 whole-brain tractograms. To do so, we mimicked the post-mortem cortex-sparing fiber dissection method recently reported (Martino et al., 2010, 2011; Sarubbo et al., 2013), which exposes the IFOF and UF stems, collecting all the fibers belonging respectively to the IFOF and UF, after meticulous removal of the cortical and U-shaped fibers. Once these two stems were isolated, we were able to perform a quantitative analysis of their cortical terminations with minimal anatomical *a priori*. The software TrackVis (Wang et al., 2007) was used to perform the virtual dissection using the streamline filtering tools to select streamline groups and to display simultaneously the streamlines and FA maps. A combination of in-house Matlab and TrackVis command line tools were used to prepare the tractograms and extract the tracts.

**FIGURE 3:**
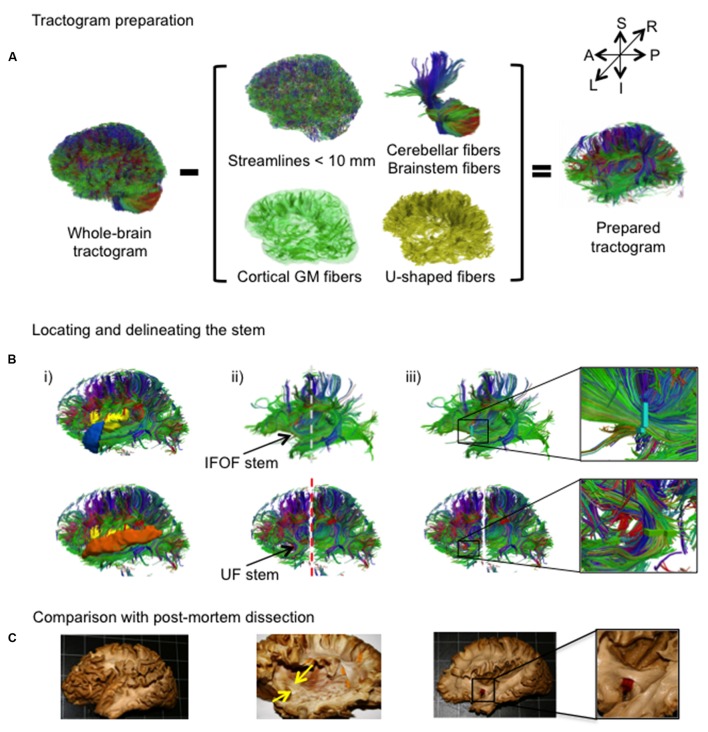
**(A,B)** The stem-based method. **(A)** The preparation of the tractogram, inspired from the postmortem dissection technique to selectively remove streamline groups not belonging to the long association pathways and **(B)** the procedure to delineate the stems of the IFOF and UF are shown on a single subject. Steps of the virtual dissection for the IFOF (**B**, first row) and UF (**B**, second row) with analogous images from a post-mortem dissection of the IFOF (**B**, third row): (i) Template regions used to remove select groups of cortical streamlines, (ii) window revealing the stem of the tract (dashed lines represent the coronal inclusion (gray) and exclusion (red) filtering slices), (iii) ROI drawn to encompass the stem of the tract, while in post-mortem dissection a red tag separates the IFOF stem from the dorsal claustrum and putamen. **(C)** Analogous steps from a post-mortem dissection of the IFOF (Sarubbo et al., 2013).

An overview of the complete virtual dissection is shown in **Figure 3**. In the first step, we removed from the whole-brain tractogram (**Figure 3A**): the extraneous streamlines shorter than 10 mm, the superficial streamlines restricted to the cortical GM region and the U-shaped streamlines passing through the SWM region. We also removed the streamlines passing through the cerebellum and the brainstem as well as the callosal streamlines passing through the inter-hemispheric fissure as they do not belong to the association pathways. In the next step, the removal of the U-shaped streamlines with terminations within the insula and the temporal pole exposed the IFOF stem (**Figure 3B**, top), while the removal of the streamlines terminating within the insula and the superior temporal gyrus exposed the UF stem (**Figure 3B**, bottom). Note that all these steps were performed using TrackVis command line tools and the ROIs of the modified JHU template. Once the stem was exposed, a ROI was manually drawn that strictly encompassed it in the same manner a paper tag is inserted under a narrow stem during *post-mortem* dissection (**Figure 3C**). The IFOF stem was drawn on a single coronal slice where the streamlines converged into a compact bundle and the width of the bundle is the smallest before projecting to the cortex. The UF stem was drawn on a single axial slice at the point where the streamlines curve downward and gather into a compact bundle, before descending to the temporal cortex. The criteria for the inclusion of streamlines within the ROI were judged with respect to: (1) their location and proximity to the core of the bundle and (2) the cohesiveness of the shape of the streamlines to those within the core of the bundle. Therefore streamlines that created a gap in the stem and deviated significantly from the shape of the streamlines in the core of the bundle were not included. Of note, a small group of streamlines following a different course than the IFOF bundle, coming rather vertically from the parietal regions, passing the lateral aspect of the IFOF stem and terminating within the external capsule, was observed in the majority of the subjects. These streamlines belonging the claustro-cortical system (Fernandez-Miranda et al., 2008; Milardi et al., 2015) were not included in the IFOF and UF tracts.

The final step was the extraction of the IFOF and UF. This was done by specifying all streamlines that pass through their respective stems from the original whole-brain tractogram. This is a key advantage of tractography while in post-mortem dissection the removal of brain tissue is definitive. We aimed to apply the least constraints possible on the analysis of the cortical termination of the two tracts. At the level of the anterior temporal cortex, the streamlines of the IFOF are directed posteriorly whereas the streamlines of the UF curve in an anterior direction to reach the temporal pole (Ebeling and von Cramon, 1992; Martino et al., 2010). Therefore, the final IFOF was extracted by including all streamlines passing through the IFOF stem while excluding those that may pass through the temporal pole. The final UF was extracted by including all streamlines passing through the UF stem and a coronal slice posterior to the external capsule was specified to exclude the streamlines oriented too posteriorly and may belong to the IFOF. **Figure 5** shows an example of both left and right IFOF and UF virtually dissected in one of the 60 subjects.

Since our focus was on the cortical terminations of these association streamlines, the projection streamlines passing through the IFOF and/or UF stems but terminating within the subcortical nuclei were not taken into account nor were those terminating within the insula whose nearby position along these stems requires a separate study. Note that we discarded the streamlines passing through a stem but stopping short of the cortical ROIs.

### Analysis of the IFOF and UF Stems

Both IFOF and UF stems were normalized to the standard Montreal Neurological Institute (MNI) space using the inverse linear and non-linear registration process. The Euclidean distance between the IFOF and UF stem center of mass coordinates was also calculated within each hemisphere as well as their respective volumes.

In order to evaluate the reliability of the stem delineation, both IFOF and UF stems were manually drawn by two different operators (GP, JH) in the 60 subjects. Their spatial matching was examined by comparing the stem volumes and the Euclidean distance between the center of mass coordinates of the stems drawn by the two operators.

### Analysis of the IFOF and UF Cortical Terminations

Once the IFOF and UF streamlines were extracted, we can describe within each subject their cortical termination territories based on macro-anatomical landmarks. Each streamline ending in one of 28 cortical regions of the JHU template was tallied to produce a measure of tract termination density for this region. The tallies were obtained using the UCLA Multimodal Connectivity Software Package (Brown et al., 2012). An individual normalized termination density score (NTDS) was obtained for each region by dividing the total number of tract streamlines ending in it by the total number of left and right tract streamlines for IFOF and UF, respectively. A first assessment was done to consider a cortical region as a termination territory of a given tract. We applied a threshold on the NTDS for each cortical region by choosing a region where the NTDSs can be considered as false positives. The precentral and superior parietal gyri were chosen as the regions containing false positives (FP_REG) for the IFOF and UF, respectively. The threshold for each tract was calculated based on the population, as follows:

$${Threshold}_{FP\_REG}=Mean\,\;\;NTDS_{FP\_REG}+(2\times S\,\tan dard\,\;\;deviation_{FP\_REG}).$$

An individual adjusted score was therefore calculated for the regions with a NTDS superior to the Threshold_FP_REG_, by the following:

$$Adjust\,\;\;NTDS_{REG}=NTDS_{REG}-Threshold_{FP\_REG}$$

The adjusted normalized density score for each region underwent the Wilcoxon Signed-Rank test, since the distributions for almost all regions were non-normal, with the null hypothesis set at ≤0. Only those surviving the Bonferroni correction (*p* < 0.0018) were considered termination territories of the tract. For each tract, the percentage of subjects with tract terminations in each of these territories was calculated.

To assess laterality effects, asymmetry indexes were calculated on the normalized density scores for each termination region using the following formula: $\frac{\left(Right-left\right)}{\left(Right+left\right)}$. Two-tailed ANOVAs using the Bonferroni correction were performed to test for significant asymmetries.

## Results

### Description of the IFOF and UF Stems

In the following, the results of the IFOF and UF stems drawn by one operator (GP) are presented (see inter-operator reliability below). The mean stem volumes (in mm^3^) of the left and right IFOFs were 82.5 ± 35.5 and 100.3 ± 47.0, respectively (**Table 1**). The mean stem volumes of the left and right UFs were 74.0 ± 33.0 and 93.9 ± 33.1, respectively. The ANOVA with Tract (IFOF, UF), Hemisphere (Left, Right) and their interaction (Tract × Hemisphere) as within-subject factors and Sex as a between-subjects factor only revealed an Hemisphere effect with both IFOF and UF stems significantly larger in the right hemisphere (*F* = 40.9, *p* < 0.0001).

**Table 1:** Summary of mean volume (in mm^3^) and mean center of mass in MNI space, for the IFOF and UF stems drawn by the two operators (JH, GP) across the 60 subjects.

		Volume	*X*	*Y*	*Z*
IFOF	Left (GP) Left (JH)	82.5 ± 35.5 88.7 ± 20.4	-30.6 ± 1.5 -31.0 ± 1.5	+0.5 ± 2.9 -1.0 ± 2.8	-8.0 ± 1.5 -9.0 ± 1.0
	Right (GP) Right (JH)	100.3 ± 47.0 91.2 ± 20.1	+31.7 ± 1.5 +31.1 ± 1.5	+0.6 ± 2.5 -0.1 ± 2.5	-7.4 ± 1.7 -8.6 ± 1.6
UF	Left (GP) Left (JH)	74.0 ± 33.0 66.7 ± 21.8	-33.3 ± 1.7 -33.8 ± 1.9	-2.4 ± 2.7 -2.7 ± 2.6	-16.2 ± 2.4 -16.8 ± 2.6
	Right (GP) Right (JH)	93.9 ± 33.1 86.1 ± 29.0	+33.6 ± 1.5 +33.1 ± 1.8	-1.2 ± 2.2 -1.7 ± 2.3	-17.2 ± 2.1 -17.6 ± 2.1

**FIGURE 4:**
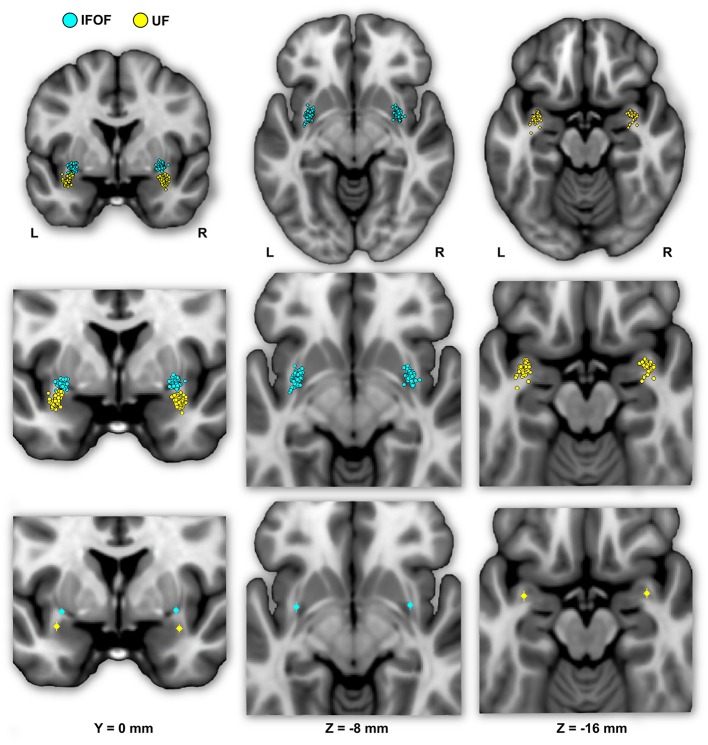
**The individual (first row) and mean (second row) locations of the IFOF and UF stem centers of mass are shown in MNI space projected on a single-subject T1 image from the on their mean coronal section (left) and axial sections (middle and right).** The IFOF stem centers of mass are located dorsally, anteriorly and medially with respect to the UF stems.

**Figure 4** shows the individual and mean center of mass locations of the IFOF and UF stems as points projected on a single-subject MNI T1 brain. The locations of the stems were highly consistent across subjects for both tracts and their relative positions within the ventral part of the external capsule are clearly distinct (**Figure 4**, coronal section). On average, the delineated IFOF stem was located between the posterior part of the putamen and the claustrum (**Figure 4**, axial section) while the delineated UF stem tended to be situated more ventrally within the external capsule. The mean center of mass coordinates of the IFOF and UF stems are presented in **Table 1**. The mean Euclidean distances between the left IFOF and UF stem centers of mass were 9.4 ± 2.7 mm and for the right IFOF and UF stems was 10.5 ± 2.1 mm, both being significantly different from 0 (Student’s *t*-test, *p* < 0.0001). The mean center of mass coordinates of the left and right UF stems were situated significantly more ventral than the respective IFOF stems in each hemisphere (mean ± standard deviation, left: 8.2 ± 2.3 mm and right: 9.7 ± 1.9 mm, *p* < 0.0001). They were also slightly but significantly more posterior (left: 2.0 ± 2.9 mm and right: 1.7 ± 2.4 mm, *p* < 0.0001) and more lateral (left: 2.7 ± 1.9 mm; right: 1.9 ± 1.6 mm, *p* < 0.0001).

### Inter-operator Reliability to Delineate the IFOF and UF Stems

The delineation of the stems was consistent between operators both in terms of volumes and mean centers of mass (**Table 1**). The mean differences of volume between operators were between 6 and 9 mm^3^ for left and right IFOF stems and between 7 and 8 mm^3^ for left and right UF stems, which corresponds approximately to a difference of one voxel (8 mm^3^) and was not significant between operators (all Student’s *t*-tests, *p* > 0.05). The mean Euclidean distances between the stems drawn by the two operators was 1.7 ± 0.8 mm and 1.9 ± 0.8 mm for the left and right IFOF stems and was 1.4 ± 1.1 mm and 1.4 ± 0.9 for the left and right UF stems, distances below the 2-mm voxel resolution at which our stems were drawn.

### Description of the Tracts

**FIGURE 5:**
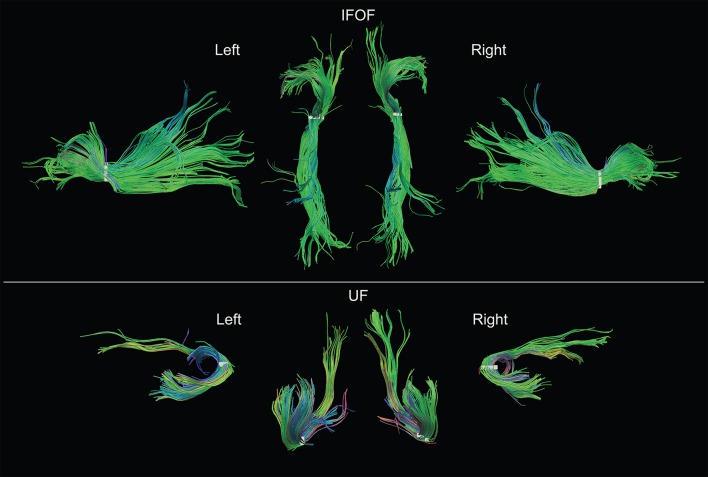
**Examples of both left and right IFOF and UF of a typical subject extracted using the stem-based anatomical virtual dissection and shown with their stems (white).** Middle top row, dorsal view of the two IFOF; middle bottom row, ventral view of the two UF.

Examples of the IFOF and UF in the left and right hemispheres of a typical subject can be seen in **Figure 5**. We present the frequency and distribution of each tract’s terminations below.

#### Frequency of Tract Terminations in Cortical Regions among 60 Subjects

For the IFOF, **Table 2A** shows the percentage of subjects with tract terminations in each of the 14 cortical regions that showed an adjusted normalized density score above 0. Anteriorly, the IFOF always projected to the inferior frontal gyrus (present in all subjects, bilaterally), almost always projected to the lateral fronto-orbital gyrus (87 and 90% for the left and right hemispheres, respectively henceforth) and less frequently projected to the medial fronto-orbital gyrus (63 and 32%) and middle frontal gyrus (60 and 55%). Projections to the superior frontal gyrus were observed in the minority of subjects (35 and 20%). Posterior IFOF termination frequencies were lower, never reaching 100%, which is related to long streamlines passing through the stem but stopping before reaching the termination territory (see Methodological Limitations in the Discussion). The most frequent posterior termination regions were the middle occipital gyrus (65 and 95%) and lingual gyrus (80 and 78%). The IFOF also terminated in the inferior occipital gyrus (30 and 77%), in the temporal lobe in the superior (73 and 45%) and middle (33 and 52%) temporal gyri, and in the parietal lobe in the superior parietal gyrus (35 and 57%). Projection to the cuneus (38 and 15%), fusiform gyrus (12 and 32%) and angular gyrus (10 and 33%) were observed in the minority of subjects.

**Table 2:** Percentage of subjects with terminations present in each region for the IFOF **(A)** and UF **(B)**.

(A)											
**IFOF**	**Frontal**					**Occipital**				**Temporal**			**Parietal**	
	**SFG**	**MFG**	**IFG**	**LFOG**	**MFOG**	**MOG**	**IOG**	**Cu**	**LG**	**STG**	**MTG**	**Fu**	**SPG**	**AG**

Left	35%	60%	100%	87%	63%	65%	30%	38%	80%	73%	33%	12%	35%	10%
Right	20%	55%	100%	90%	32%	95%	77%	15%	78%	45%	52%	32%	57%	33%

**(B)**										

**UF**	**Frontal**					**Temporal**					**Limbic**
	**MFG**	**IFG**	**LFOG**	**MFOG**	**RG**	**T. pole**	**STG**	**MTG**	**ITG**	**Fu**	**Ent**

Left	12%	37%	88%	100%	83%	100%	100%	98%	88%	37%	77%
Right	60%	35%	98%	100%	93%	100%	100%	97%	92%	43%	78%

*A connection to a region was considered present for a subject if the termination density score for the region was above the threshold determined using the false positive region for each tract.*

For the UF, **Table 2B** shows the percentage of subjects with tract terminations in each of the 11 cortical regions that showed an adjusted normalized density score above 0. Anteriorly, the UF always terminated in the medial fronto-orbital gyrus (present in all subjects, bilaterally), almost always projected to the lateral fronto-orbital (88 and 98%) and rectus (83 and 93%) gyri. Projections to the middle (12 and 60%) and inferior (37 and 35%) frontal gyri were also observed but with less frequency. Posteriorly, the UF always terminated in the temporal pole (present in all subjects, bilaterally) and superior temporal gyrus (present in all subjects, bilaterally) and almost always in the middle temporal gyrus (98 and 97%). The UF also frequently projected to the inferior temporal gyrus (88 and 92%) and entorhinal gyrus (77 and 78%). Projections to the fusiform gyrus were observed in the minority of subjects (37 and 43%).

#### Distribution of Tract Terminations

**FIGURE 6:**
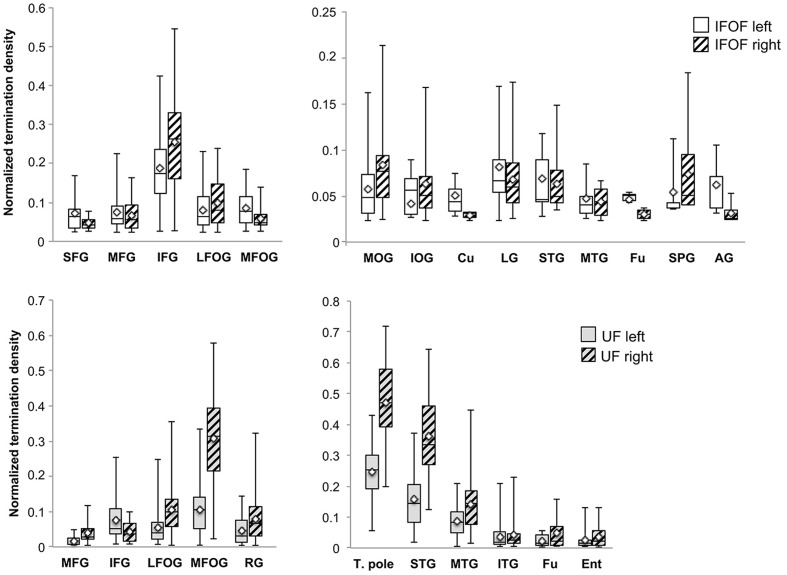
**The normalized termination density scores of each anterior and posterior cortical region for the IFOF and UF are shown as box plots with their means (diamonds).** Abbreviations (see **Figure 1**).

A description of the distribution of the tract terminations can be given based on the quantity of streamlines from the reconstructed streamlines terminating within each cortical region. **Figure 6** shows the normalized termination densities of each region as box plots for the IFOF and UF tracts.

Anteriorly, the IFOF terminates predominantly in the inferior frontal gyrus (NTDS: 0.19 and 0.26 for the left and right, respectively henceforth) with minor branches projecting to the lateral (0.08 and 0.10) and medial fronto-orbital (0.09 and 0.06) and superior (0.05 and 0.04) and middle frontal gyri (0.07 and 0.06). Note that these NTDS do not account for 100% of the tract streamlines as a result of the streamlines stopping prematurely anteriorly and posteriorly. Given the larger proportion of posteriorly ‘broken’ streamlines, posterior densities are likely to be underestimated. Posteriorly the largest proportions of IFOF projections are in the middle occipital (0.06 and 0.08) and lingual (0.08 and 0.07) gyri, followed by the superior temporal gyrus (0.07 and 0.06) with widely distributed minor branches in the occipital lobe in the inferior occipital gyrus (0.04 and 0.06) and cuneus (0.05 and 0.03), in the temporal lobe in the middle temporal (0.05 and 0.04) and fusiform (0.05 and 0.03) gyri, and in the parietal lobe in the superior parietal (0.05 and 0.07) and angular (0.06 and 0.03,) gyri.

For the UF, the anterior projections are most predominant in the medial fronto-orbital gyrus (0.10 and 0.31) and distributed across the lateral fronto-orbital (0.05 and 0.11), rectus (0.05 and 0.08) and inferior frontal (0.08 and 0.04) gyri with the fewest projections in the middle frontal gyrus (0.02 and 0.04). Posteriorly, the UF projects in a graded manner with the largest portion in the temporal pole (0.25 and 0.47), followed by the superior temporal (0.16 and 0.36) and middle temporal (0.09 and 0.14) gyri and with minor branches in the inferior temporal (0.04 bilaterally), fusiform (0.03 and 0.05) and entorhinal (0.03 and 0.05) gyri.

#### Asymmetry of Tract Terminations in Individual Regions

**FIGURE 7:**
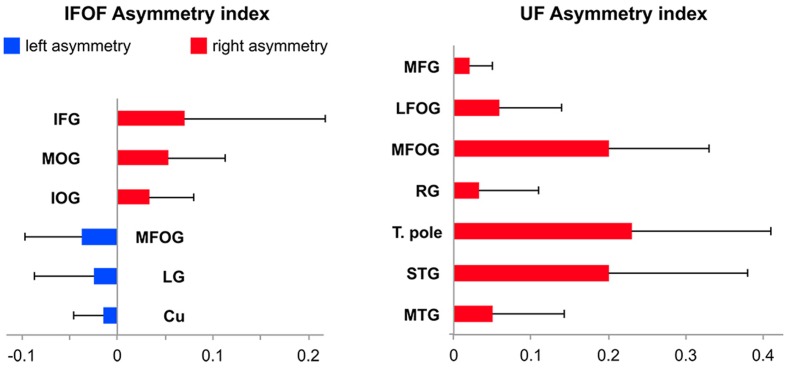
**Mean tract termination density asymmetry indexes of the IFOF and UF showing significant lateralization for the number of streamlines**.

Among the 14 IFOF terminal regions, significant lateralization patterns were observed differently for its anterior and posterior projections (all Bonferroni-corrected *p*-values < 0.004). A rightward lateralization of the inferior frontal projections and leftward lateralization of medial fronto-orbital projections were observed for anterior IFOF terminations (**Figure 7**). Among its posterior projections, significant rightward lateralization of middle occipital, inferior occipital projections were observed. Significant leftward lateralization was observed in the medial occipital areas in the cuneus and lingual gyrus (**Figure 7**).

Among the 11 UF terminal regions (all Bonferroni-corrected *p*-values < 0.005), significant rightward lateralization was observed in lateral and medial fronto-orbital gyri, in the middle frontal gyrus and rectus gyrus (**Figure 7**). Posterior UF terminations were significantly rightward lateralized in the temporal pole, superior and middle temporal gyri.

## Discussion

The present anatomical stem-based virtual dissection allowed us to efficiently isolate and extract both IFOF and UF. Previous dissection studies have noted that the distinction between these two tracts is difficult due to the intermingling of their fibers (Ebeling and von Cramon, 1992; Kier et al., 2004). However, they are clearly distinct at the level of the stem and can be separated by a notable cleavage line (Curran, 1909; Kier et al., 2004; Martino et al., 2010; Sarubbo et al., 2013). This was confirmed in this study with the UF stem situated significantly ventrally, posteriorly and laterally to the IFOF stem (**Figure 4**) consistent with two studies on the relative topographies of tracts in this area (Ebeling and von Cramon, 1992; Kier et al., 2004).

**FIGURE 8:**
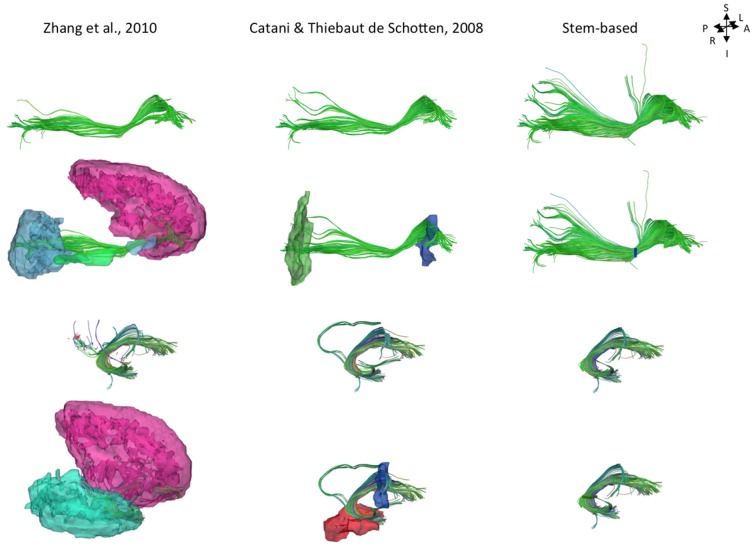
**Examples of the IFOF and UF tracts in a single subject segmented using different ROI-based methods: automatic template ROI sets (left; Zhang et al., 2010), obligatory passages method (middle; Catani and Thiebaut de Schotten, 2008) and the stem-based method (right, only whole anterior-to-posterior cortical streamlines are shown).** The ROIs are shown in the second and fourth rows (exclusion ROIs are not shown).

While current tract segmentation approaches rely heavily on *a priori* knowledge, using set-of-ROIs strategies that place the ROIs around their known terminal areas or areas of passage (Catani and Thiebaut de Schotten, 2008; Zhang et al., 2010), our stem-based virtual dissection results in more extensive IFOFs and UFs. **Figure 8** compares the different IFOFs and UFs of a single subject obtained by applying current set-of-ROIs methods and the present stem-based virtual dissection. Note the parietal and superior frontal projections of the IFOF present in the stem-based extraction that are absent in the other IFOFs.

### Methodological Limitations

Before discussing the results of both IFOF and UF terminations, we address some noticeable limitations that are due to the tractography method used to obtain the whole-brain tractograms. We used the diffusion tensor model (FACT; Mori et al., 1999), which is well known for its limited ability to resolve crossing fiber configurations within a voxel. Thus areas of intersecting/overlapping/bordering pathways will greatly affect the tracking process and cause it to stop, producing broken streamlines. In our case, the streamlines start within a cortical region, pass through the stem, but stop before reaching their final cortical destination. This is especially noticeable for the IFOF, which presents the longest streamlines with the highest chance to cross other tracts. This is likely to account for the lack of IFOF projections to the superior frontal gyrus and relatively low occurrence of posterior projections for example in the middle and superior occipital gyri. *Post-mortem* dissection studies have noted considerable overlap between the infero-superior course of the IFOF and antero-posterior course of the arcuate fasciculus near the inferior and middle frontal regions (Martino et al., 2010; Sarubbo et al., 2013). Similarly the posterior projections of the IFOF need to pass through the inferior longitudinal fasciculus within the temporal lobe in order to reach the middle occipital gyrus. Moreover the large variability observed in the frequency of projections among subjects in these areas are likely to be due to such limitations and thus their densities may also be underrepresented. Note that, in future studies, using more advanced tractography methods such as higher-order modeling (Descoteaux et al., 2009; Tournier et al., 2012) and anatomically constrained tracking (Girard et al., 2014) will help to prevent broken streamlines and enable the study of their whole connectional anatomies.

Nonetheless, thanks to the stem-based virtual dissection our approach allowed us to: (1) reproduce the consensus findings regarding IFOF and UF tract projections, and (2) provide clear evidence for more extensive projections beyond their conventional definitions (see **Figure 1B** for a revised definition of both tracts).

### Cortical Terminations of the Stem-Based IFOF

Our study first confirmed frontal IFOF terminations within the inferior frontal and lateral and medial fronto-orbital gyri corresponding to the conventional IFOF definition, but also in the superior and middle frontal gyri in line with recent studies (Sarubbo et al., 2013; Caverzasi et al., 2014). We showed that the IFOF’s primary branch is clearly the inferior frontal subcomponent followed by the orbito-frontal subcomponent, with about equal density across the superior frontal and middle frontal subcomponents. These are consistent with the proportions reported in (Croxson et al., 2005) that used probabilistic tractography. The prominence of the inferior frontal and orbito-frontal terminations may explain why these regions are consistently observed in the literature. It could also be that the superior frontal streamlines densities may be underrepresented given the limitations of the tractography method mentioned above.

Posteriorly, we observed occipital projections to the middle and inferior occipital gyri also consistent with the conventional definition of the IFOF, as well as additional terminations in the occipital (cuneus and lingual gyrus), temporal (superior and middle temporal gyri, fusiform gyrus) and parietal (superior parietal and angular gyri) cortices. This corresponds to one of the earliest descriptions of the IFOF by Curran (1909) who described it as *“… a large associating bundle of fibers uniting, as it name indicates, the occipital with the frontal lobe. It also contains fibers, which join the frontal lobe with the posterior part of the temporal and parietal lobes.*” In terms of posterior density distribution, we showed that the IFOF projects mainly to the occipital lobe (especially the middle occipital and lingual gyri, with minor projections to the inferior occipital gyrus and cuneus), with minor projections to the temporal lobe (superior and middle temporal and fusiform gyri) as well as to the parietal lobe (superior parietal and angular gyri), consistent with Curran’s IFOF description.

The medial occipital cortex (cuneus and lingual gyrus) are not often cited as a termination territory of the IFOF possibly be due to a lack of anatomical specificity in some of the earlier descriptions of the tract, with its first mention appearing late in the literature (Crosby et al., 1962). Projections to the lingual gyrus were observed in almost all subjects consistent with Caverzasi et al. (2014), however, Martino et al. (2010) reported no projections to the lingual gyrus and instead considered them as part of the optic radiations. Projections to the cuneus were infrequent in the right hemisphere (present in 15 *vs.* 38% in the left hemisphere) showing the opposite pattern of Caverzasi et al. (2014) who reported more subjects in the left than in the right hemispheres. These differences may be due in part to the difference in templates used to register the terminations (they used the Freesurfer Desikan-Kyliany atlas whereas we used the JHU atlas). Projections to the inferior occipital gyrus were observed, twice less in the left than in the right hemisphere, though it was reported in all subjects in a dissection study (Martino et al., 2010). This may be due to possible crossings with other prominent tracts (inferior longitudinal, vertical occipital fasciculi) in the occipital lobe, especially for the left hemisphere. In contrast, the superior occipital gyrus was not significant as a termination territory (Martino et al., 2010). This may also be due to technical limitations arising from the tractography method since it is farther and may require tracking through areas of crossings.

In the temporal lobe, the IFOF projects to the superior and middle temporal gyri and to a lesser extent to the fusiform gyrus, confirming dissection studies (Crosby et al., 1962; Martino et al., 2010). Neither the superior temporal nor the middle temporal gyri were reported previously (Caverzasi et al., 2014).

We were also able to observe parietal projections specifically in the superior parietal gyrus and to a lesser extent in the angular gyrus. As shown **Figure 8**, projections to the parietal lobe are systematically excluded using the method of Zhang et al. (2010) and are likely to be missed using the method of Catani and Thiebaut de Schotten (2008) that use ROIs delineated around the frontal and occipital lobes, as was the case in Sarubbo et al. (2013). There is converging evidence for the existence of fronto-parietal connections for the IFOF from *post-mortem* (Curran, 1909; Martino et al., 2010) and tractography studies (Mori et al., 2005; Caverzasi et al., 2014). We observed superior parietal IFOF terminations in the majority of the subjects consistent with dissection [Martino et al. (2010) reported parietal connections in 9 out of 14 hemispheres], and less frequently in the angular gyrus in contrast to Caverzasi et al. (2014) who reported it in 100% of subjects.

### Asymmetry of Cortical IFOF Terminations

We observed a rightward lateralization of projections to the lateral frontal and occipital areas (inferior frontal, middle and inferior occipital gyri), and a leftward lateralization of projections to medial frontal and occipital areas (superior frontal, medial fronto-orbital, cuneus and lingual gyri) (**Figure 7**). Interestingly, these lateral/medial lateralization patterns are consistent with the different superficial/deep subcomponents of the IFOF defined by Sarubbo et al. (2013). These different lateralization patterns within the IFOF subcomponents may partly explain the conflicting reports regarding the asymmetry of the IFOF (Thiebaut de Schotten et al., 2011; Forkel et al., 2014). They also suggest distinct anatomo-functional roles for a right lateralized lateral/superficial subcomponent and a left lateralized medial/deep subcomponent.

### Cortical Terminations of the Stem-Based UF

We confirmed anterior UF terminations mainly within the medial and lateral orbito-frontal areas, and minor branches in the middle and inferior frontal and rectus gyri consistent with dissection and tractography studies (Klinger and Gloor, 1960; Ebeling and von Cramon, 1992; Thiebaut de Schotten et al., 2012; Von Der Heide et al., 2013). The present results are also consistent with the proportions reported in Croxson et al. (2005).

Posterior UF terminations were predominantly observed within the temporal pole consistent with Ebeling and von Cramon (1992), followed by the superior and middle temporal gyri, and then by fusiform and entorhinal gyri. We did not observe any projection to the cingulate gyrus, neither to the amygdala in agreement with (Dejerine and Dejerine-Klumpke, 1895; Ebeling and von Cramon, 1992) but in contrast to (Klinger and Gloor, 1960; Croxson et al., 2005; Thiebaut de Schotten et al., 2012). These discrepancies may be due to the existence of adjacent tracts, such as the amygdalo-temporo or amygdala-prefrontal pathways (Klinger and Gloor, 1960; Kim and Whalen, 2009) which may have been sometimes misattributed to the UF. Importantly, projections to the nuclei of the amygdala reported in Ebeling and von Cramon (1992) refers to the entorhinal cortex and should not be mistaken for the amygdala proper as is often done (Schmahmann and Pandya, 2006; Kim and Whalen, 2009).

Beyond the temporal pole, we showed evidence of terminations posterior to the vertical plane passing through the anterior commissure in the superior, middle and inferior temporal gyri. Note that the hook-shaped pattern of these posterior temporal streamlines looks more like UF than IFOF streamlines, which led us to consider them as belonging to the UF. This was in line with our goal to follow as close as possible the neurodissectionist gesture. As such, we consistently observed a cleavage zone within the ventral portion of the external capsule between the UF fibers turning anteriorly and inferiorly to the temporal lobe (including the superior, middle and inferior temporal gyri posterior to the temporal pole) and the IFOF fibers going deeply and dorsally (Martino et al., 2010; Sarubbo et al., 2013).

### Asymmetry of Cortical UF Terminations

The UF projections were mainly right lateralized. Hemispheric asymmetry in the UF has been observed for both volume and diffusion metrics but there are inconsistencies in the directionality of such asymmetry of the UF. Previous diffusion studies reported a rightward lateralized UF (Highley et al., 2002; Park et al., 2004; Thomas et al., 2015), however, asymmetry in the opposite direction has also been shown (Kubicki et al., 2002; Hasan et al., 2009) and some did not find any asymmetry in the UF (Thiebaut de Schotten et al., 2011). Such inconsistencies underline the need for further investigation, possibly with more advanced tractography methods.

## Conclusion

In this study, we applied an original anatomical ROI-based method to virtually dissect two association tracts, IFOF and UF, in a large group of subjects. By delineating anatomically both IFOF and UF stems, we minimized the constraints on the tract terminations and observed far more extensive projections than their conventional definitions (**Figure 1B**). These previously unconsidered projections of the IFOF and UF need to be integrated into their structural definitions and considered when interpreting the multi-functional roles of these tracts. In addition, the quantitative information available through diffusion imaging provides not only tract-specific but branch-specific measures of the tracts providing better specificity that can be correlated with behavioral measures or patient populations. Such studies paired with complementary techniques such as direct electrical brain stimulation studies (Duffau, 2015), will be an important step in understanding their functional roles that will be relevant in the clinical setting.

## Author Contributions

Designed, acquired and pre-processed the BIL&GIN database including diffusion data: GP, FC, LZ, EM, GJ, MJ, BM, NT-M, and LP. Analyzed the data: JH, GP, SS, and LP. Wrote the paper: JH and LP.

## Conflict of Interest Statement

The authors declare that the research was conducted in the absence of any commercial or financial relationships that could be construed as a potential conflict of interest.
